# Combined effects of maximal oxygen uptake and glucose status on mortality: The Prospective KIHD cohort study

**DOI:** 10.1111/sms.14135

**Published:** 2022-02-13

**Authors:** Sudhir Kurl, Pirjo Hakkarainen, Ari Voutilainen, Eija Lönnroos

**Affiliations:** ^1^ Institute of Public Health and Clinical Nutrition University of Eastern Finland Kuopio Finland

**Keywords:** cardiorespiratory fitness, cardiovascular mortality, diabetes, noncardiovascular mortality, prediabetes

## Abstract

**Objective:**

To examine the combined effects of cardiorespiratory fitness (CRF) and prediabetes or diabetes on cardiovascular and noncardiovascular mortality.

**Patients and methods:**

This prospective study evaluated a population‐based cohort of 1562 men aged 42–60 years at baseline (1984–1989). We utilized maximal oxygen uptake (VO_2max_) for assessing aerobic capacity and CRF in the cohort and stratified participants into six groups according to both their glucose status (diabetes, prediabetes, or no diabetes) and whether they were below‐ or above‐median VO_2max_. Deaths in the cohort were recorded till December 31 2016. Cox regression was used to calculate hazard ratios (HR) with 95% confidence intervals (CI) for cardiovascular and noncardiovascular mortality. Smoking, alcohol consumption, BMI, blood pressure, cholesterol, diagnosis of ischemic heart disease, and socioeconomic status served as covariates in the mortality analyses.

**Results:**

During the follow‐up (mean 24.2 years), 341 men died from cardiovascular and 468 men from noncardiovascular causes. When compared to men with no diabetes and above‐median VO_2max_, the presence of either diabetes (HR = 4.10, 95% CI: 2.27–7.40) or prediabetes (HR = 2.10, 95% CI: 1.18–3.73) combined with below‐median VO_2max_ increased the risk of cardiovascular death. Noncardiovascular mortality was increased by low oxygen uptake in men with prediabetes (HR = 2.24, 95% CI: 1.30–3.84), and among men with diabetes, the increase was not statistically significant (HR = 1.99, 95% CI: 0.91–4.32).

**Conclusions:**

Cardiorespiratory fitness modifies the risk of death related to prediabetes and diabetes. This highlights the importance of CRF assessment and interventions to support the uptake of regular physical activity among aging men with disturbed glucose metabolism.

**Key Points**

1. The combined effects of cardiorespiratory fitness (CRF) and glucose metabolism on mortality are not clear.
2. Maximal oxygen uptake (VO_2max_) was used to assess aerobic capacity and CRF in a population‐based cohort of middle‐aged (42–60 years) men. CRF modified the risk of death related to prediabetes and diabetes during the follow‐up of 24 years.
3. Low oxygen uptake increased the risk of dying of a cardiovascular disease irrespective of the glucose metabolism status at midlife. Noncardiovascular mortality was also increased in those men with low oxygen uptake independently of whether they exhibited normal glucose metabolism or prediabetes.
4. Interventions should be targeted at improving CRF among aging men with disturbed glucose metabolism.

## 1 INTRODUCTION

Impaired fasting glucose (IFG) and impaired glucose tolerance (IGT) are conditions in which an individual's glucose levels are above the normal range but not high enough to be diagnostic for diabetes.^1^ These prediabetic conditions may occur separately or in combination, and both are associated with an increased risk of a progression to type 2 diabetes.^2^ It is well known that diabetes increases the risk of cardiovascular mortality,^3^ but associations with noncardiovascular deaths are less clear. Prediabetic conditions contribute to cardiovascular morbidity though less prominently as is the case for diabetes.^3^, ^4^ Furthermore, findings concerning the impact of prediabetic conditions on mortality are inconsistent.^5^

Physical activity and the resulting cardiorespiratory fitness (CRF) are associated with several health benefits including a lower risk of diabetes, cardiovascular morbidity, and mortality.^6^, ^7^ Current evidence indicates that the majority of the risk reduction is achieved by 75–150 min of vigorous or 150–300 min of moderate‐intensity physical activity.^7^ When compared with inactivity, significant health benefits can be achieved with as little as 15 min of daily exercise of moderate intensity.^8^

In this study, we investigated the combined effects of CRF and prediabetes or diabetes on mortality of middle‐aged men. We used maximal oxygen uptake (VO_2max_) for assessing aerobic capacity and CRF in the population‐based male cohort. Our working hypothesis was that prediabetes and diabetes would represent an increased risk for cardiovascular and noncardiovascular mortality and that interactions with CRF could modify these risks.

## 2 MATERIALS AND METHODS

### 2.1 Study population

**FIGURE 1:**
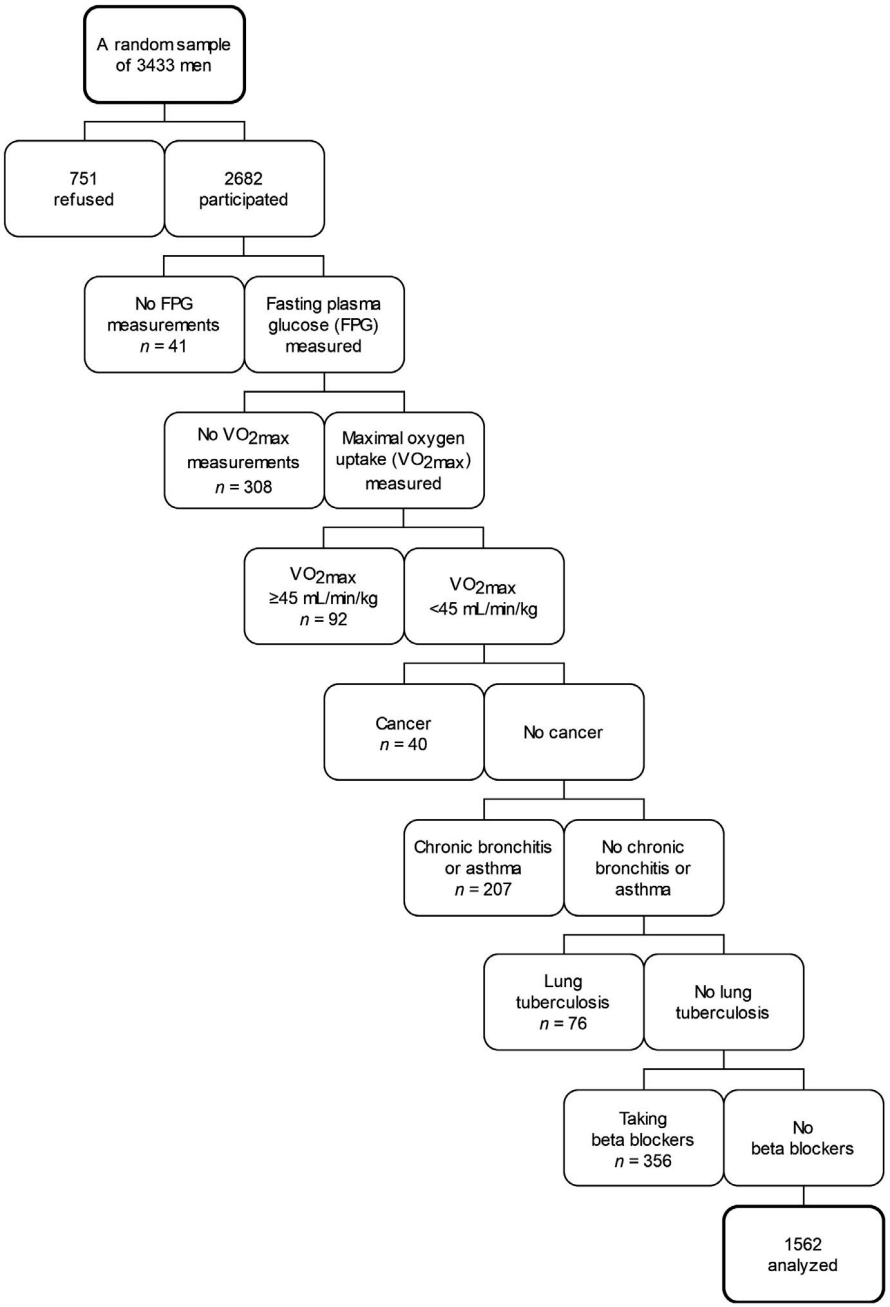
Population‐based sample, participants, and exclusions (boxes on the left side)

The Kuopio Ischaemic Heart Disease Risk Factor Study (KIHD) is an ongoing population‐based cohort study of a randomly selected sample of 3433 men aged 42–60 years and residing in the city of Kuopio or its surrounding rural communities in 1984.^9^ Of those who were invited, 2682 (83%) participated in the study. This study focused on the KIHD subpopulation (*n* = 1562) in whom glucose status and maximal oxygen uptake (VO_2max_) had been measured and relevant confounding conditions excluded (Figure 1). Baseline examinations were conducted between March 20, 1984, and December 5, 1989, and the deaths in the cohort were recorded till December 31, 2016. The KIHD protocol was approved by the Research Ethics Committee of the University of Kuopio, and each participant gave written informed consent.

### 2.2 Assessment of diabetes, prediabetes, and normoglycemia

Fasting plasma glucose (FPG) was measured using the glucose dehydrogenase method (Merck, Darmstadt, Germany) after proteins had been precipitated with trichloroacetic acid. Subjects meeting the following criteria were defined as patients with diabetes: either having regular treatment with an oral hypoglycemic agent, insulin therapy, or having treatment only with diet while also having an FBG level >6.9 mmol/L.^10^ Prediabetes among nondiabetic subjects was defined according to the recommendations of the American Diabetes Association, and FPG within the range from 5.6 mmol/L to 6.9 mmol/L.^11^ FPG < 5.6 mmol/L was considered as no diabetes.^11^, ^12^

### 2.3 Assessment of CRF and VO_2max_

VO_2max_ is the gold standard for assessing aerobic capacity and CRF.^13^, ^14^ When measuring the participants’ VO_2max_, we used a maximal symptom‐limited exercise tolerance test on an electrically braked bicycle ergometer and direct gas exchange analysis at the study baseline in 1984–1989.^15^, ^16^ For 407 men in the present KIHD subpopulation, the testing protocol comprised of a three‐minute warm‐up at 50 W followed by a step‐by‐step increase in the workload by 20 W per minute (Tunturi EL 400 bicycle ergometer, Turku, Finland), respiratory gas exchange measured by the mixing chamber method (Mijnhardt Oxycon 4 analyzer, Odijk, the Netherlands), and VO_2max_ expressed as average of values recorded over a 30‐s period. The remaining 1155 men were tested with 20 W per minute linear increase in the workload (Medical Fitness Equipment 400 L bicycle ergometer, Mearn, the Netherlands) and with the breath‐by‐breath method (MGC 2001 analyzer, Medical Graphics, St. Paul, Minn., USA) with VO_2max_ being expressed as the average of values recorded over 8 s. VO_2max_ was defined as the highest value for, or the plateau of, oxygen uptake.

The measure of leisure time physical activity was based on self‐report and covered a 12‐month period. The participants were asked to report the frequency (number of sessions/month), average duration (hours and minutes/session), and type of each activity performed. The intensity of leisure time physical activity was calculated and expressed in metabolic equivalents of oxygen consumption (MET).^15^

### 2.4 Assessment of covariates

The baseline values of the following variables were used as covariates in the analyses of mortality: smoking, alcohol consumption, body mass index (BMI), systolic blood pressure (SBP), total cholesterol (TC), socioeconomic status (SES), diagnosis of ischemic heart disease, employment status, and marital status. The variables were selected on the basis of previous research, their confounding potential,^5^, ^8^, ^12^, ^17^, ^18^, ^19^ and avoiding unnecessary adjustment.

The lifelong exposure to smoking (cigarette pack‐years) was estimated as the product of the number of years smoking and the number of tobacco products smoked daily at the time of examination.^11^, ^17^ Alcohol consumption was assessed using the Nordic Alcohol Consumption Inventory.^18^ BMI was computed as the ratio of weight in kilograms to the square of height in meters. The diagnoses of chronic conditions, including ischemic heart disease, were checked at the baseline examinations by the internist. Resting blood pressure was measured between 8:00 and 10:00 a.m. with a random‐zero sphygmomanometer.^17^ The cholesterol contents of serum lipoprotein fractions and triglycerides were measured enzymatically (Boehringer Mannheim, Mannheim, Germany). SES was defined with an index that combined data on education, occupation, income, and material standards of living and housing conditions.^19^ A higher score in the SES index indicated a lower socioeconomic status. In addition, current employment (yes/no) and marital status (living alone/single or cohabiting/married) were self‐reported.

Subjects (*n* = 679) with conditions that might strongly affect measurements of VO_2max_ and/or outcome of death were excluded from the analyses, that is, cancer, chronic bronchitis or asthma, lung tuberculosis, and the use of beta blockers (Figure 1). From the main analyses, we excluded also subjects (*n* = 92) with a superior maximal oxygen uptake (≥45 mL/min/kg), as they do not represent the general middle‐aged Finnish male population^20^ but, for example, correspond to young male soldiers.^21^ To ensure, this exclusion did not affect main findings, and additional Cox regressions with men whose VO_2max_ was ≥45 mL/min/kg were conducted.

### 2.5 Classification of deaths

Statistics Finland annually links the date of death and primary, immediate, and intermediate causes of death to KIHD subjects. The causes of death are accordant with diagnoses of the International Classification of Diseases (ICD).^22^ Cardiovascular deaths refer to ICD‐10 codes I00–I99 and ICD‐9 codes 390–459. Noncardiovascular deaths refer to all other causes of death. In this study, we stratified deaths according to the primary cause of death.

### 2.6 Statistical analyses

We used Cox regression to predict the risk of cardiovascular and noncardiovascular mortality in the study population. In addition to glucose status and CRF (VO_2max_), the explanatory variables were the following seven continuous variables (age, smoking, alcohol consumption, BMI, SBP, TC, and SES) and three categorical variables (ischemic heart disease, employment status, and marital status). In the analysis, we stratified the subjects into six groups according to their glucose status (diabetes, prediabetes, or no diabetes) and whether they were below‐ or above‐median VO_2max_. Median VO_2max_ in the whole cohort was 31.26 mL/min/kg. In the mortality analyses, the reference category was always men without diabetes and with above‐median VO_2max_. IBM^®^ SPSS^®^ Statistics Version 25 served as a platform for the analyses.

## 3 RESULTS

Table 1 presents the baseline characteristics of 1562 subjects analyzed in this study. By the end of the year 2016 (mean follow‐up 24.2 SD 8.1 years), a total of 809 men had died, 341 due to cardiovascular and 468 due to noncardiovascular causes. The most common primary causes for cardiovascular deaths were chronic ischemic heart disease (*n *= 115), acute myocardial infarction (*n *= 95), and cerebral infarction (*n *= 28). Correspondingly, malignant neoplasms *(n* = 231*)* and Alzheimer's disease and other dementias (*n *= 95) were the most common primary causes of noncardiovascular deaths.

**TABLE 1:** Characteristics of subjects distributed into six groups according to their glucose status and median (31.26 mL/min/kg) of maximal oxygen uptake (VO_2max_)

Variable	Diabetes and below‐median VO_2max_	Diabetes and above‐median VO_2max_	Prediabetes with below‐median VO_2max_	Prediabetes with above‐median VO_2max_	No diabetes and below‐median VO_2max_	No diabetes and above‐median VO_2max_	Total
*n*	25	13	47	19	709	749	1562
Cardiovascular deaths (*n*)	15	4	17	5	192	108	341
Noncardiovascular deaths (*n*)	7	5	18	5	246	187	468
Age (years)	54.4 (4.0)	51.7 (4.6)	52.9 (5.0)	49.7 (5.9)	53.7 (4.7)	51.4 (5.2)	52.5 (5.1)
Alcohol consumption (g week^−1^)	133.0 (163)	77.6 (158)	84.5 (127)	124.0 (131)	83.9 (150)	64.0 (101)	75.7 (129)
Body mass index	29.9 (4.0)	28.0 (3.3)	30.8 (4.3)	26.8 (2.6)	27.4 (3.6)	25.7 (2.7)	26.8 (3.4)
Systolic blood pressure (mmHg)	144 (19.6)	140 (19.4)	148 (20.2)	135 (16.0)	135 (17.0)	132 (15.1)	134 (16.5)
Total cholesterol (mmol l^−1^)	5.8 (1.2)	5.9 (0.8)	5.9 (1.1)	6.1 (0.9)	5.9 (1.0)	5.8 (1.0)	5.9 (1.0)
Socioeconomic status score (0−25)	12.8 (5.4)	10.4 (3.5)	13.2 (4.1)	10.0 (4.6)	12.6 (5.0)	11.6 (5.1)	12.1 (5.0)
Smoking (cigarette packs day^−1^ times years of smoking)	7.7 (15.8)	0.4 (1.7)	6.0 (11.8)	10.7 (23.2)	11.7 (19.6)	6.3 (13.6)	8.8 (16.9)
Ischemic heart disease (yes %)	28.0	7.6	31.9	15.8	22.3	10.1	16.6
Employment status (unemployed %)	44.0	0	27.7	5.26	30.2	14.7	22.3
Marital status (unmarried %)	12.0	38.5	21.3	15.8	16.1	10.3	13.6
Self‐reported leisure‐time physical activity							
Duration (h week^−1^)	7.3 (4.9)	7.3 (4.7)	7.0 (6.0)	5.7 (4.9)	7.5 (8.1)	7.6 (6.1)	7.5 (7.0)
Intensity ‐MET*, mean (SD)	4.9 (1.3)	5.1 (1.3)	4.2 (0.9)	4.7 (0.8)	4.4 (1.0)	4.8 (1.2)	4.5 (1.1)

Note

For continuous variables, numbers indicate mean and standard deviation.

* MET, Metabolic equivalent of oxygen consumption.

### 3.1 Risk of cardiovascular death

**FIGURE 2:**
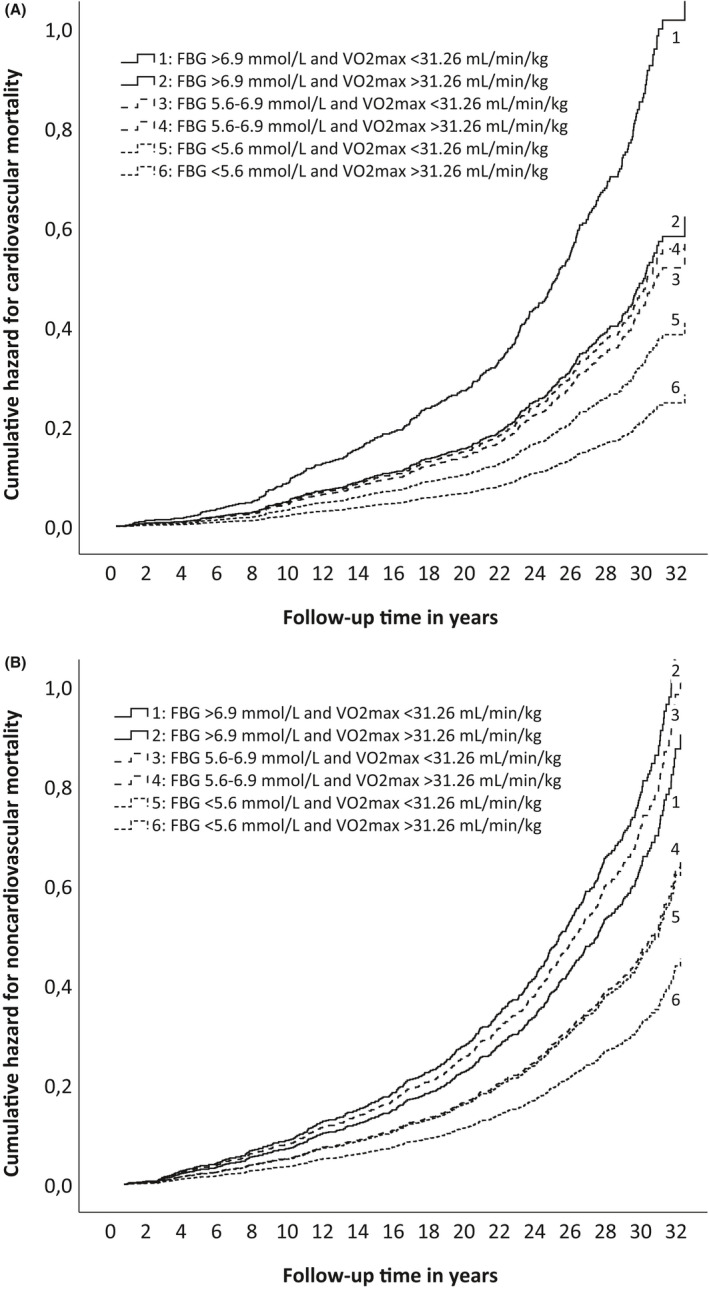
Cumulative hazard for cardiovascular and noncardiovascular mortality among subjects distributed into six groups according to their fasting blood glucose (FBG) level and whether they had below‐ or above‐median maximal oxygen uptake (VO_2max_)

Men with diabetes and below‐median VO_2max_ had four times higher cardiovascular mortality when compared to men with no diabetes and above‐median VO_2max_ (Table 2, Figure 2). Having prediabetes and below‐median VO_2max_ doubled the risk of cardiovascular death. In addition, the below‐median VO_2max_ increased cardiovascular mortality by 55% among men with no diabetes. Both diabetes and prediabetes combined with above‐median VO_2max_ increased the hazard ratio for cardiovascular death, but these findings were not statistically significant. In the Cox regression models, increasing age, BMI, SBP, TC, and smoking and being unemployed or unmarried all were factors statistically significantly contributing to cardiovascular mortality. When 92 men with the highest maximal oxygen uptake (≥45 mL/min/kg) were included in the analyses, HRs slightly changed but statistical significance remained unchanged.

**TABLE 2:** Hazard ratios (HRs) for risk factors of cardiovascular mortality

Factor	HR	95% CI	*p*	*d* ^1^	HR^5^
Diabetes with below‐median VO_2max_ ^2^, ^3^	4.10	2.27−7.40	<.001	1.101	3.70
Diabetes with above‐median VO_2max_ ^2^, ^4^	2.35	0.85−6.45	.097	0.666	2.34
Prediabetes with below‐median VO_2max_ ^2^, ^3^	2.10	1.18−3.73	.011	0.578	2.04
Prediabetes with above‐median VO_2max_ ^2^, ^4^	2.24	0.91−5.55	.079	0.632	2.02
No diabetes with below‐median VO_2max_ ^2^, ^3^	1.55	1.20−2.00	.001	0.344	1.46
Age (years)	1.08	1.05−1.11	<.001	0.064	1.09
Alcohol consumption (g week^−1^)	1.00	1.00−1.00	.339	0	1.00
Body mass index (kg/m^1^)	1.03	1.00−1.06	.046	0.026	1.04
Systolic blood pressure (mmHg)	1.01	1.01−1.02	<.001	0.013	1.02
Total cholesterol (mmol L^−1^)	1.15	1.04−1.27	.004	0.114	1.16
Socioeconomic status score (0−25)	1.02	0.99−1.04	.095	0.015	1.02
Smoking (cigarette packs day^−1^ times smoking years)	1.01	1.01−1.02	<.001	0.013	1.02
Ischemic heart disease (yes vs. no)	1.12	0.85−1.46	.408	0.089	1.14
Unemployed (yes vs. no)	1.38	1.06−1.79	.014	0.255	1.40
Unmarried (yes vs. no)	1.50	1.12−1.99	.005	0.317	1.50

^1^ Cohen's $d=\ln\left(\mathrm{HR}\right)\times \frac{\sqrt{6}}{\pi }$ (34).

^2^ Reference category is no diabetes with above‐median VO_2max_.

^3^ Below‐median VO_2max_ <31.26 mL/min/kg.

^4^ Above‐median VO_2max_ >31.26 mL/min/kg.

^5^ Analyses also included 92 men with VO_2max_ ≥45 mL/min/kg.

### 3.2 Risk of noncardiovascular death

Having prediabetes and below‐median VO_2max_ doubled the risk of noncardiovascular death when compared to no diabetes and above‐median VO_2max_ (Table 3, Figure 2). Among men with no diabetes, the below‐median VO_2max_ increased noncardiovascular mortality by 41%. Diabetes with below‐ or above‐median VO_2max_ seemed to increase the hazard ratio for noncardiovascular death, but the finding was not statistically significant. In the Cox regression models, increasing age, alcohol consumption, SES, and smoking and being unmarried were factors significantly associated with noncardiovascular mortality. When 92 men with the highest maximal oxygen uptake (≥45 mL/min/kg) were included in the analyses, HRs for noncardiovascular death slightly changed but no notable effect on statistical significance occurred.

**TABLE 3:** Hazard ratios (HRs) for risk factors of noncardiovascular mortality

Factor	HR	95% CI	*p*	*d* ^1^	HR^5^
Diabetes with below‐median VO_2max_ ^2^, ^3^	1.99	0.91−4.32	.082	0.537	1.75
Diabetes with above‐median VO_2max_ ^2^, ^4^	2.44	0.99−6.01	.051	0.698	2.70
Prediabetes with below‐median VO_2max_ ^2^, ^3^	2.24	1.30−3.84	.003	0.629	2.17
Prediabetes with above‐median VO_2max_ ^2^, ^4^	1.45	0.59−3.54	.413	0.291	1.31
No diabetes with below‐median VO_2max_ ^2^, ^3^	1.41	1.14−1.74	.001	0.270	1.38
Age (years)	1.06	1.04−1.09	<.001	0.051	1.07
Alcohol consumption (g week^−1^)	1.00	1.00−1.00	<.001	0.001	1.00
Body mass index (kg/m^1^)	0.97	0.94−1.00	.150	−0.017	0.99
Systolic blood pressure (mmHg)	1.00	0.99−1.00	.643	0.001	1.00
Total cholesterol (mmol L^−1^)	0.98	0.90−1.08	.806	−0.009	0.99
Socioeconomic status score (0−25)	1.04	1.01−1.06	<.001	0.031	1.04
Smoking (cigarette packs day^−1^ times smoking years)	1.01	1.01−1.02	<.001	0.012	1.02
Ischemic heart disease (yes vs. no)	0.89	0.69−1.14	.365	−0.090	0.91
Unemployed (yes vs. no)	1.23	0.98−1.55	.073	0.163	1.24
Unmarried (yes vs. no)	1.66	1.30−2.12	<.001	0.398	1.63

^1^ Cohen's $d=\ln\left(\mathrm{HR}\right)\times \frac{\sqrt{6}}{\pi }$ (34).

^2^ Reference category is no diabetes with above‐median VO_2max_.

^3^ Below‐median VO_2max_ <31.26 mL/min/kg.

^4^ Above‐median VO_2max_ >31.26 mL/min/kg.

^5^ Analyses also included 92 men with VO_2max_ ≥45 mL/min/kg.

## 4 DISCUSSION

If a man has a low oxygen uptake, this increases his risk of dying of cardiovascular disease irrespective of his glucose metabolism status at age of 42–60 years. However, the increase in cardiovascular mortality was highest in men with diabetes. Noncardiovascular mortality was also increased by low oxygen uptake in those men with prediabetes or with no diabetes. Interestingly, the level of VO_2max_ did not statistically significantly modify noncardiovascular mortality in men with diabetes. The novelty of our study is that it demonstrates the combined effects of CRF and glucose metabolism on both cardiovascular and noncardiovascular mortality.

It is well known that diabetes is associated with reduced survival. A meta‐analysis of 102 prospective studies reported that diabetes doubled the risk of cardiovascular death.^3^ This concerned also male populations. The presence of diabetes increased cardiovascular and all‐cause mortality in Swedish men aged 48 years,^23^ among the US male physicians aged 40–84 years^24^ and in a British male cohort aged 52–74 years.^25^ Similar findings have been previously reported from this KIHD cohort of middle‐aged Finnish men, where diabetes increased the risk of sudden cardiac death and all‐cause mortality.^12^ The impact of diabetes on cardiovascular and all‐cause mortality has been confirmed also among middle‐aged Finnish women.^26^, ^27^ To the best of our knowledge, previous studies have not investigated the combined effects of diabetes and oxygen uptake on mortality. In our study, diabetes combined with low oxygen uptake increased the risk of cardiovascular death, and although the risk seemed to decrease if the man had a high oxygen uptake, it did not eliminate his diabetes‐related risk.

There is increasing evidence that also prediabetes increases mortality. A meta‐analysis of cohort studies including both male and female populations reported that prediabetes increased all‐cause mortality by 13%–32%.^5^ For example, in the Hoorn study, all‐cause and cardiovascular mortality increased among 50‐ to 75‐year‐old persons with prediabetes.^28^ In the Australian Diabetes, Obesity and Lifestyle Study, IFG increased mortality by 1.6‐fold and IGT by 1.5‐fold in persons aged 25 years and over.^29^ In contrast, the Cardiovascular Health Study conducted among older North Americans reported that prediabetes was not associated with increased mortality^30^; this was also the conclusion of a German study of individuals aged 55–74 years.^31^ In male cohorts, prediabetes has been found to be a significant risk factor for cardiovascular mortality,^23^ sudden cardiac death,^12^ coronary heart disease mortality,^32^ and all‐cause mortality.^12^, ^23^ In our study, prediabetes combined with low oxygen uptake significantly increased the risks of cardiovascular and noncardiovascular deaths among men.

It is evident that sufficient physical activity and good CRF may prevent prediabetic conditions and their conversion to diabetes.^1^ Physical activity has an important and recognized role in the treatment of diabetes.^1^, ^33^ Furthermore, physical activity and good CRF have been shown to reduce cardiovascular morbidity and mortality.^6^, ^7^ However, the associations with cancer mortality are less clear. A recent meta‐analysis reported that the linear association between sedentary behavior and cancer mortality was nonsignificant and remained unaffected after adjustment for physical activity.^34^ Evidence from epidemiological studies suggests that physical activity may protect from the development of Alzheimer's disease (AD) or delay the disease onset.^35^ Even among older adults who are at a high risk of dementia, good CRF was associated with better cognitive functioning during two‐year follow‐up.^36^ In addition, evidence from three large multidomain dementia prevention trials suggested that individuals at an increased risk for dementia may benefit from multidomain lifestyle interventions, which include a physical activity component.^37^ Thus, it does seem that low physical activity and diabetes are both risk factors for AD^38^ and AD is associated with an increased risk of death.^39^

Cardiorespiratory fitness^40^, ^41^ and VO_2max_ ^18^ are important predictors of survival. Every 1 MET increase in physical fitness is associated with a 10–20% risk reduction in all‐cause mortality.^40^, ^42^, ^43^ An important feature is that CRF does not need to be exceptionally high to provide significant protection. A CRF level of 5–8 METs has been associated with a substantial increase in survival,^41^ with greater protection associated with good CRF^40^, ^41^ The mechanisms explaining why a good CRF contributes to reduced cardiovascular morbidity and improved survival even years after its assessment are not well understood. CRF and VO_2max_ are influenced by several factors, including age, sex, genetics, smoking, and the presence of clinical or subclinical (cardiac, pulmonary, and skeletal muscle) diseases; nevertheless, habitual physical activity remains the most important determinant of CRF.^44^ Our important finding is that VO_2max_ provides prognostic information on the survival of middle‐aged men with prediabetes or diabetes. Moreover, the prognostic value seems to be independent of other known cardiovascular risk factors, such as smoking, hypertension, overweight, and total cholesterol. This confirms the finding that better CRF reduces the risk of premature death even among individuals with unfavorable risk profiles,^40^, ^42^ including prediabetes and diabetes. This indicates that men with prediabetes or diabetes may benefit from higher physical fitness irrespective of their other risk factors.

The strength of our prospective study is that we have a representative population‐based sample of middle‐aged men, a high participation rate, and complete data on deaths. The coverage and quality of the national register data on occurrence and causes of deaths have been shown to be extremely high.^45^ Furthermore, we had reliable data on baseline health status and we were able to control for the confounding effects of several conventional cardiovascular and sociodemographic risk factors in our analyses. The prospective nature of our study, comprehensive baseline measurements, and exclusions made for the present analysis enabled us to manage and avoid the biases attributed to reverse causality.

There are limitations that deserve some consideration. First, our study probably had insufficient statistical power for assessing the combined effects of diabetes and CRF. The number of men with diabetes was rather low and the VO_2max_ categorized subgroups even smaller. Second, one measurement of VO_2max_ cannot rule out some variation with time or during the follow‐up. However, VO_2max_ is a gold standard for measuring cardiorespiratory fitness, and it is also associated with cardiovascular and all‐cause mortality.^13^, ^19^ Other applicable methods to assess physical activity and fitness are based on self‐reporting and direct measurements. The self‐reported physical activity is often used in population studies, but it may result in inaccuracy.^46^ Direct measurements of physical activity such as accelerometers were not available when the KIHD study was designed. Third, the total duration of prediabetes or diabetes was not known, a factor that has an impact on the appearance of complications, morbidity, and mortality.^3^, ^4^ Fourth, prediabetes refers to both IFG and IGT. In our study, prediabetes was assessed by the level of FPG. An oral glucose tolerance test, which may reveal IGT, was not used. Fifth, we examined an ethnically and genetically homogenous population of the same gender, which limits the generalizability of our findings. However, there is no evidence that the predictive value of CRF would be less important among female subjects.^43^ The combined effects of CRF and glucose metabolism on mortality warrant further studies in female populations.

## 5 PERSPECTIVE

It is known that diabetes and prediabetes increase mortality.^3^, ^5^ We found that CRF modifies the risk of death related to prediabetes and diabetes. Our study suggests that not only men with type 2 diabetes but also prediabetic men with reduced CRF have an increased risk of death. CRF seems to have independent prognostic value in survival in men with prediabetes or diabetes. It is evident that sufficient physical activity and good CRF may prevent prediabetic conditions and their conversion to diabetes,^1^ similarly as they have been shown to reduce cardiovascular morbidity and mortality.^6^, ^7^ This highlights the importance of performing a CRF assessment and indicates that individuals with prediabetes or diabetes are an important target group for physical activity interventions and should be encouraged to participate in regular physical activity programs.

## CONFLICT OF INTEREST

Sudhir Kurl, Pirjo Hakkarainen, Ari Voutilainen, and Eija Lönnroos declare that they have no conflict of interest.

## AUTHOR CONTRIBUTIONS

The study concept and design: SK, PH, AV, and EL. Statistical analyses: AV. Drafting: SK. Critical revision: AV, PH, and EL. All authors have approved the final version of the manuscript.

## Data Availability

The data that support the findings of our study are available upon a reasonable request from the Institute of Public Health and Clinical Nutrition, University of Eastern Finland.
